# Population dynamics and genomic characterization of *Salmonella* Infantis reveal poultry as a major reservoir of antimicrobial resistance genes and pESI megaplasmid

**DOI:** 10.1128/spectrum.04020-25

**Published:** 2026-04-23

**Authors:** Sabin Poudel, Jinquan Wang, Dianna Bourassa

**Affiliations:** 1 Department of Poultry Science, Auburn University 708995, Auburn, Alabama, USA; Universita degli Studi di Bari Aldo Moro, Valenzano, Bari, Italy

**Keywords:** *Salmonella *Infantis, AMR, pESI-carriage, source, location

## Abstract

*Salmonella* enterica serovar Infantis is one of the emerging multidrug-resistant serovars causing foodborne illness to humans. Here, we analyzed the genome sequences of 16,157 *S*. Infantis isolated globally to illustrate the population dynamics, risk of antimicrobial resistance, and pESI megaplasmid carriage rates. The population structure revealed that, irrespective of the source and location of isolation, greater than 95% of isolates were ST-32. *S*. Infantis from ST-32 was further analyzed using the ResFinder and PlasmidFinder databases in the Abricate platform. Population dynamics and odds ratios (OR) of antimicrobial resistance (AMR) across sources and continents were analyzed using logistic regression, with multiple comparisons assessed using Tukey’s HSD test and Benjamini-Hochberg false discovery rate (FDR) correction applied to both global and pairwise *P*-values. The significance level was set at 0.05. Analysis of ST-32 *S*. Infantis isolates revealed that the risk of harboring AMR genes was significantly higher in isolates collected from poultry compared to other sources, despite having variation in the number of genes and the magnitude of the odds ratio. Interestingly, irrespective of the source of isolation, almost all *S*. Infantis isolates possess *aac (6*′*)-Iaa* genes, whereas the most prevalent AMR genes among poultry-sourced *S*. Infantis were *tet(A*), followed by *sul1 and ant (3*′*)-Ia*. Additionally, the carriage rate of the pESI-plasmid was higher in poultry (87.90%), followed by human isolates (26.98%). In conclusion, our work provides an insight into multilocus sequence typing (MLST), AMR, and carriage rates of the pESI-like plasmid among *S*. Infantis isolated globally from diverse isolation sources.

**IMPORTANCE:**

Foodborne illness caused by *Salmonella* remains a major public health concern. Among >2,500 known *Salmonella* serovars, *S*. Infantis ranks among the top 10 serovars causing human illness. The significance of *S*. Infantis is further amplified by its ability to harbor and disseminate antimicrobial resistance (AMR) genes mediated by pESI-like megaplasmid. Therefore, understanding the population dynamics and assessing the risk of AMR gene carriage in *S*. Infantis isolates collected from diverse sources and geographic locations are critical. The findings from this analysis help identify high-risk reservoirs and track current AMR trends, enabling the development of streamlined treatment strategies and data-driven interventions aimed at limiting the global spread of multidrug-resistant *S*. Infantis.

## INTRODUCTION

Nontyphoidal *Salmonella* remains the leading foodborne illness-causing bacteria in the United States, with an estimated 1.35 million infections annually leading to approximately 19,336 hospitalizations and 378 deaths (1). The economic impact caused by the nontyphoidal *Salmonella* outbreaks was predicted to be $4.1 billion annually (2), and the economic burden due to *Salmonella* contamination from poultry sources was estimated at $2.8 billion (3). Nontyphoidal *Salmonella* infection in humans causes gastroenteritis; however, in most instances, infection results in self-limited gastroenteritis (4) and subsides within a week without any medical intervention (5). However, the infection may become fatal to young and elderly people (6) and the immunocompromised, requiring medical treatments (7). In some cases, infection due to invasive nontyphoidal *Salmonella* may result in bacteremia, leading to meningitis (8), osteomyelitis (9), and endocarditis (10), requiring antibiotic treatments. Additionally, the emergence of multidrug-resistant *Salmonella* serovars continues to pose a significant public health threat, specifically the resistance of *Salmonella* against ciprofloxacin, azithromycin, and cephalosporin antibiotics that are critically important for treating human illness (11).

**Fig 1:**
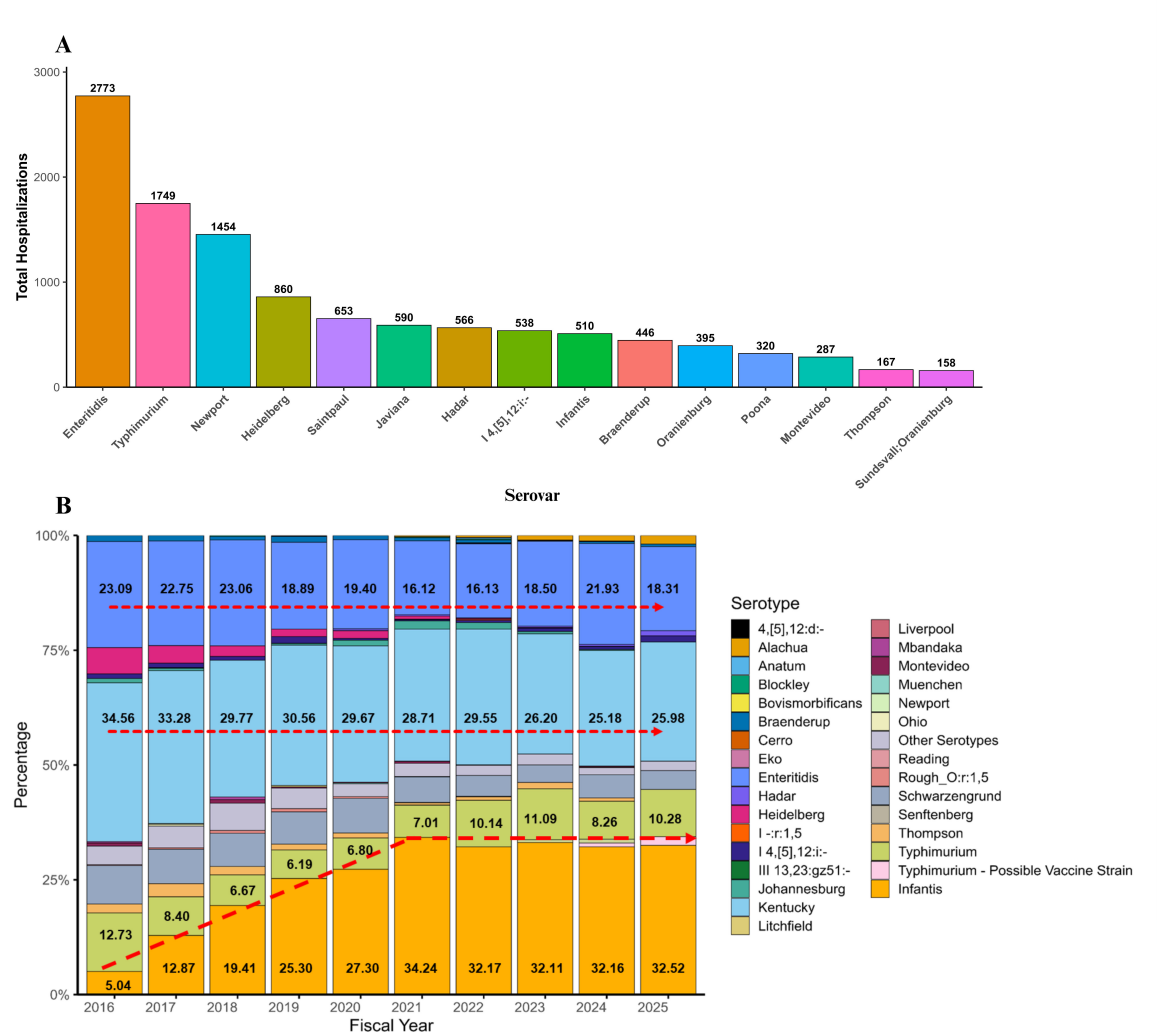
*Salmonella* serovar distribution in human infections and Food Safety and Inspection Service (FSIS) chicken isolates. (**A**) Top 15 *Salmonella* serovars causing human hospitalizations in the United States over time according to the National Outbreak Reporting System (NORS) data. (**B**) Yearly proportion of *Salmonella* serovars isolated from chicken in FSIS surveillance, showing changes in serovar prevalence over time according to FSIS data.

Poultry is commonly identified as a source of foodborne *Salmonella* illness, with an attribution estimated to be 19.0% (3). The dynamics of *Salmonella* serovar detection in poultry samples across the United States has shifted over time from serovar Kentucky to Infantis (Fig. 1). During the fiscal years of 2016–2017, *S*. Kentucky was the historically predominant serovar with an approximately 30% isolation rate, whereas more recent data suggest *S*. Infantis as the emerging serovar (12) (Fig. 1). Similarly, a surge in the prevalence of *S*. Infantis has been identified in the European Union in recent years (13) as *S*. Infantis accounted for 33.9% isolates from a food-animal source (14) (The European Union One Health 2021). A zoonoses report predicted that 95% of *S*. Infantis isolates were strictly related to a broiler source (13). Additionally, the increased *S*. Infantis prevalence in poultry carcasses might also be reflected in increasing trends of human salmonellosis.

Although there might be several hypotheses that can explain the increased prevalence of *S*. Infantis among poultry, one possibility might be due to the implementation of targeted intervention strategies against *S*. Enteritidis and *S*. Typhimurium, either via the development of vaccination or any other chemical intervention (14). Another possibility for increased *S*. Infantis prevalence might be attributed to its genetic arsenal as it possesses a pESI-like megaplasmid, which enhances its ability to survive harsh conditions as well as helps the acquisition and transmission of resistant and virulent genes (15, 16). Despite increasing concerns about the global spread and public health threats posed by resistant *S*. Infantis, there is limited information on its resistance patterns and distribution. Therefore, the objective of this study was to analyze the global dynamics of *S*. Infantis and assess the distribution and odds of the occurrence of AMR genes and plasmid types among isolates collected from diverse sources and geographic regions.

## MATERIALS AND METHODS

### Data acquisition and bioinformatic analysis

The sequences of *Salmonella* Infantis and associated metadata were obtained from the NCBI and Pathogen Finder database (15 Nov 2024). A total of 16,157 sequences were obtained, and serovar identification was conducted (File S1; Table S1). Only *Salmonella enterica* subsp. *enterica* serovar Infantis was included in this study, while other rough or incompletely typed Infantis-designated strains were not considered for further analysis. The obtained genome sequences serovar were confirmed prior to downstream MLST v2.23.0 (https://github.com/tseemann/mlst) analysis (17). Since sequence type (ST) 32 accounts for approximately 95% of the samples, in our further analysis, we only focused on this ST-32 of *S*. Infantis. Therefore, ST-32 (File S1; Table S2) was further analyzed using Abricate v.1.01 (18) (https://github.com/tseemann/abricate) for the presence of antimicrobial resistance (AMR) genes using ResFinder and Plasmid finder (19) with minimum identity 90% and minimum coverage 80%, accessed on 24 November 2024. Thus, the generated AMR genes and plasmid genes’ presence/absence matrix was further analyzed. Detection of the presence of pESI-like plasmids in *S*. Infantis genome sequences was conducted by constructing a local database using 14 pESI plasmid indicator genes (File S1; Table S3) (20). *S*. Infantis possessing pESI *repA* and any five additional targets among tested 14 pESI plasmid indicators with ≥95% identity and coverage were considered pESI-positive isolates, and the carriage rate was determined per metadata category (File S1; Table S3).

### Metadata arrangements and statistical analysis

**Fig 2:**
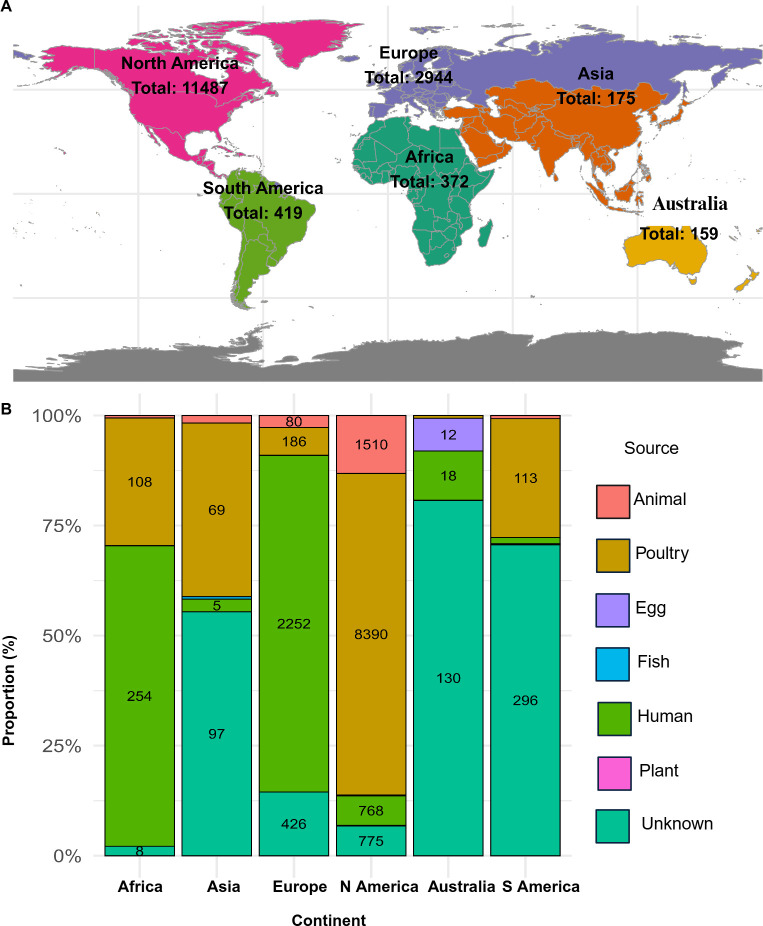
Geographical distribution and source composition of *S*. Infantis isolates included in this study. (**A**) World map showing the total number of *S*. Infantis isolates analyzed per continent. The map was created using the GIS/world map package in R. (**B**) Stacked bar plot showing the proportion of different isolates per source per continent. Absolute isolate counts per source are labeled.

The number of *S*. Infantis genomes analyzed in this study was obtained from diverse locations, with North America having the highest number of sequences and Australia having the least (Fig. 2A). For statistical analysis, due to the variation in the keywords in the metadata of submitted files, the sources were re-categorized into animal, poultry, human, plant, fish, and table egg (Fig. 2B; File S1; Table S4). Additionally, within the poultry sources, the submitted categories were re-categorized to compare the poultry carcass, poultry ceca, hatching egg, and poultry house environment (File S1; Table S5). AMR genes and plasmids identified via Abricate were also re-categorized (File S1; Tables S6 and S7), and then data were binary-transformed. The transformed binary data set was further utilized for the calculation of summary statistics and data visualization.

Population dynamics and odds ratios (OR) of AMR genes, plasmid types, and pESI-like plasmid presence/absence results across sources and continents were analyzed using a generalized linear model with binomial family, and a logit link was used to stabilize variance (21). A likelihood ratio test was used to detect the significant difference of sources and continents using the ANOVA and *post hoc* pairwise comparisons performed with the emmeans package using Tukey’s HSD. Multiple comparisons were further corrected using the Benjamini-Hochberg false discovery rate (FDR) method for both global and pairwise tests. Odds ratios and 95% confidence intervals were estimated using the logistic regression model (binomial generalized linear model) for each gene across each categorical grouping variable (File S2). The significance level was set at 0.05. Data were visualized using a dual heatmap, where only significant pairwise comparisons (FDR < 0.05) were plotted along with their odds ratio, following removal of redundant reciprocal comparisons.

## RESULTS

### Multilocus sequence typing (MLST) overview

A total of 16,157 sequences of *S*. Infantis were obtained from the NCBI database and PathogenFinder database. Among available sequences, 96.56% (15,602/16,157) of sequences fell under ST-32, which was followed by the ST-603 0.91% (148/16,157). While analyzing the STs of *S*. Infantis isolated in the USA, similar trends were observed with 99.15% (10,850/10,943) ST-32. Additionally, while analyzing the STs based on the source of the isolation, a similar pattern of domination of ST-32 was observed, with 96.44% (1,600/1,659) in animal isolates, 99.33% (8,877/8,936) in poultry isolates, and 93.41% (3,303/3,536) in human isolates.

### Global prevalence and dynamics of AMR genes

**Fig 3:**
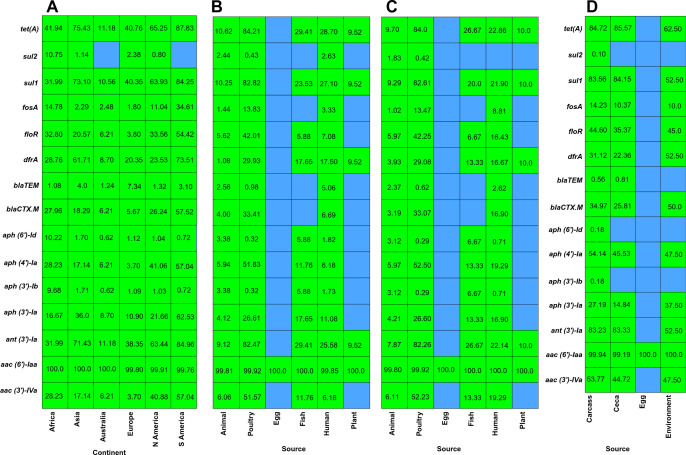
Prevalence of antimicrobial resistance genes among *S*. Infantis isolated from diverse sources and locations. (**A**) Heatmap showing the prevalence of AMR genes across continents. (**B**) Heatmap showing the global prevalence of AMR genes among *S*. Infantis isolated from diverse sources. (**C**) Heatmap showing the prevalence of AMR genes among *S*. Infantis isolated within USA (US-only source) from diverse sources. (**D**) Heatmap showing the prevalence of AMR genes among *S*. Infantis isolated from diverse sources within poultry.

In this study, *S*. Infantis ST32 was isolated around the world, and their genome sequences available in the NCBI database were analyzed for the presence of AMR genes using the ResFinder database. The prevalence percentage of top 15 AMR genes predicted by ResFinder is presented in Fig. 3. The comprehensive frequency and percentage data for all detected AMR genes by continent, source, US-only source, and poultry isolates are summarized in File S1 (Tables S8 to S11). Among various AMR genes predicted by ResFinder, *aac (6*′*)-Iaa* was the most common gene among the *S*. Infantis, with an almost 100% prevalence irrespective of the source of isolation and geographical location (Fig. 3A). Following *aac (6*′*)-Iaa, tet(A*) had the highest prevalence for all continents, whereas isolates collected from South America (87.83%) had a much higher prevalence compared to Asia (73.43%), Africa (40.94%), Australia (11.18%), Europe (40.76%), and North America (65.25%). Similarly, *sul1* and *ant (3)-Ia* genes also follow a similar trend of prevalence among the continents, with the highest prevalence in South America, followed by Asia, North America, Europe, Africa, and Australia (Fig. 3A).

While comparing the AMR genes’ prevalence among the various sources of isolation globally, *S*. Infantis isolated from poultry sources had a higher prevalence *of tet(A)* (84.21%)*, sul1* (82.82%), *ant (3*′*)-Ia* (82.47%), *aac (3*′*)-IVa* (51.57%), *floR* (42.01%), *bla_CTX_*._M_ (33.41%), and *dfrA* (29.93%) (Fig. 3B). Similar trends were observed among the *S*. Infantis isolated within the USA for the prevalence of AMR genes based on the source of isolation as we observed poultry source isolates had *tet(A)* (84.0%)*, sul1* (82.61%), *ant (3*′*)-Ia* (82.26%), *aph (4*′*)- Ia (26.60%*), *floR* (42.25%), *bla_CTX_*._M_ (33.07%), and *dfrA* (29.08%) (Fig. 3C). Additionally, while analyzing the AMR genes’ dynamics within the poultry sources, poultry carcass and cecal isolates had similar trends in having AMR genes, whereas environmental isolates had a slightly different prevalence. Similar to the other categories, in poultry sources too, *aac (6*′*)-Iaa* had an almost 100% prevalence, followed by *ant(3*′*)-Ia, tet(A), sul1, aph (4*′*)-Ia, floR*, and *bla_CTX.M_* having a higher prevalence (Fig. 3D).

### Prevalence of plasmid types and pESI-like megaplasmid

**Fig 4:**
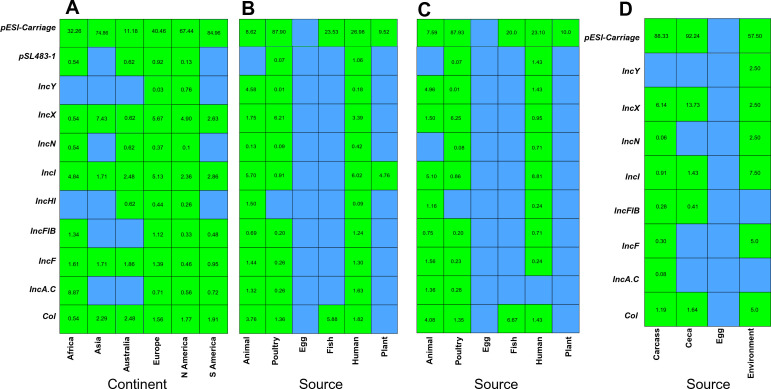
Prevalence of different plasmid types among *S*. Infantis isolated from diverse sources and locations. (**A**) Heatmap shows the prevalence of plasmid types across major continents. (**B**) Heatmap showing the global prevalence of plasmid types among *S*. Infantis isolated from diverse sources. (**C**) Heatmap showing the prevalence of plasmid types among *S*. Infantis isolated within the USA (US-only source) from diverse sources. (**D**) Heatmap showing the prevalence of plasmid types among *S*. Infantis isolated from diverse sources within poultry.

The prevalence percentage of the top 10 plasmids along with pESI-like plasmids predicted by PlasmidFinder is presented in Fig. 4. The comprehensive frequency and percentage data for all detected plasmids by continent, source, US-only source, and poultry isolates are summarized in (Tables S12 to S15). Among various plasmids predicted by the PlasmidFinder, the occurrence of the *IncA*.C plasmid was higher (8.87% among the African isolates, whereas the IncX plasmid was most common among the Asian (7.43%), European (5.67%), North American (4.90%), and South American (2.63%) isolates. The percentage of the IncFIB plasmid, which is associated with carrying AMR and virulence genes, was higher among African isolates (1.34%), followed by Europe (1.12%) (Fig. 4A). Plasmid IncX was the most common among poultry isolates worldwide at 6.21%, followed by the Col plasmid (1.36%), whereas plasmid IncI at 6.02% was common in human isolates, followed by the IncX plasmid (3.39%). The occurrence of the IncFIB plasmid among the *S*. Infantis isolates was higher in human (1.24%) isolates compared to other sources of isolation (Fig. 4B). Similar trends of plasmid prevalence were observed between the sources worldwide and US-only *S*. Infantis, and the occurrence of IncFIB plasmids was higher in animals (0.75%), followed by humans (0.71%) and poultry (0.20%) (Fig. 4C), whereas within the poultry sources, the occurrence of the IncX plasmid among cecal isolates was higher compared to that of other sources. The prevalence of *S. Infantis* isolated from poultry environments with a higher prevalence of *Col, IncI, IncN*, and *IncY* plasmid was compared to that from the other poultry sources (Fig. 4D).

In addition to the plasmid types, the carriage rate of a pESI-like plasmid among *S*. Infantis was analyzed. The overall carriage rate of the pESI-like plasmid among the *S*. Infantis was 65.36%. While comparing the carriage rate of pESI among the different locations, isolates from South America (84.96%) had the highest carriage rate, followed by isolates from Asia (74.86%) and North America (67.44%). Isolates collected from Australia (11.18%) had the lowest carriage rate of the pESI plasmid in *S*. Infantis (Fig. 4A). Among the different sources of isolation, *S*. Infantis isolated from the poultry source had the highest carriage rate of 87.9%, followed by humans at 26.98% (Fig. 4B). The trends of the pESI carriage rate among different sources within the USA were also similar to worldwide trends as poultry (87.93%) had the highest carriage rate, followed by humans (23.1%) (Fig. 4C). Whereas while comparing the pESI plasmid carries rate within the poultry sources, isolates collected from poultry ceca had a 92.24% carriage rate, followed by carcass (88.33%), and the environmental isolates carriage rate was 57.50%.

### Occurrence of AMR genes: continental odds ratio analysis

**Fig 5:**
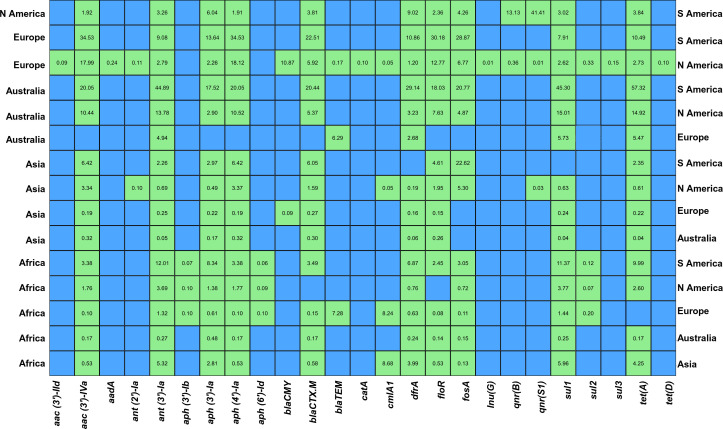
Odds ratio of antimicrobial resistance genes among *S*. Infantis across geographical location. The heatmap displays the odds ratios (OR) of AMR genes between continental pairs. The reference continent is shown on the y-axis (left), the comparison continent on the y-axis (right), and the list of AMR genes is shown on the x-axis (bottom). If the OR value > 1, it indicates a higher prevalence of AMR in the continent compared to the reference, and an OR value < 1 indicates a higher prevalence in the reference continent. Green tiles with annotated OR indicate statistically significant pairs (FDR-adjusted *P* < 0.05), whereas blank blue tiles represent nonsignificant comparisons where the FDR-adjusted *P* > 0.05.

The impact of the source of isolation on odds of AMR gene presence in *S*. Infantis is summarized in Fig. 5, whereas the corresponding analytical results are provided in File S3. To assess the difference in AMR gene odds ratio among continents (Africa, Asia, Australia, Europe, North America, and South America), we performed analysis considering each continent as reference, ensuring that each continent was compared with every other continent. While comparing the Australian isolates with other continental isolates, Australian isolates had notably lower odds of finding AMR genes in *S*. Infantis than other continents. For the aminoglycoside resistance genes, Australia had significantly lower odds specifically for *aac (3)-IVa*, *ant(3*′*)-Ia*, and *aph(4*′*)-Ia*, compared to Asia, Africa, North America, and South America; however, it has lower odds only for *ant(3*′*)-Ia* compared to Europe. In the case of the beta-lactam resistance genes, Australia had significantly lower odds only for *bla_TEM_* compared to Europe and *bla_CTX_._M_*, compared to Africa, Asia, North America, and South America. Similarly, Australia had significantly lower odds for *dfrA* and *tet(A*) compared to other continents (Fig. 5; File S3).

Current results show that Europe had lower odds of detecting AMR genes for several genes compared to Asian, South American, and African (except for *ant (3*′*)-Ia*, *bla_TEM_*, cmlA1, and *sul1*) isolates. However, Europe had higher odds of detecting AMR compared to Australia. Additionally, compared to North American isolates, odds of detecting AMR genes for aminoglycoside (*aac (3)-IId, aadA, and ant (2)-Ia*), beta-lactamase (*bla_TEM_*), chloramphenicol (*catA and cmlA1*), lincosamide (*InuG*), colistin (*mcr9*), quinolones (*qnrs1*), sulfonamide (*sul2 and sul3*), and tetracycline (*tetD*) were significantly higher in Europe (FDR-adjusted *P* < 0.05). However, some of the genes from the same category’s odds were significantly higher (FDR-adjusted *P* < 0.05) in North America than in Europe: aminoglycosides [*aac (3)-IVa, ant (3)-Ia, aph (3)-Ia, and aph (4)-Ia*], beta-lactamase (*bla_CMY_ and bla_CTX-M_*), float pathway (*dfrA*), fluoroquinolones (*flor and fosA*), sulphasomidine (*sul1*), and tetracycline [*tet(A) and tet(B*)].

The odds of detecting AMR genes in North America was lower compared to that of South America for all tested genes (FDR-adjusted *P* < 0.05). However, there were mixed results for Africa, Asia, and Europe (Fig. 5; File S3).

### Source-based occurrence of AMR gene: Odds ratio assessment

**Fig 6:**
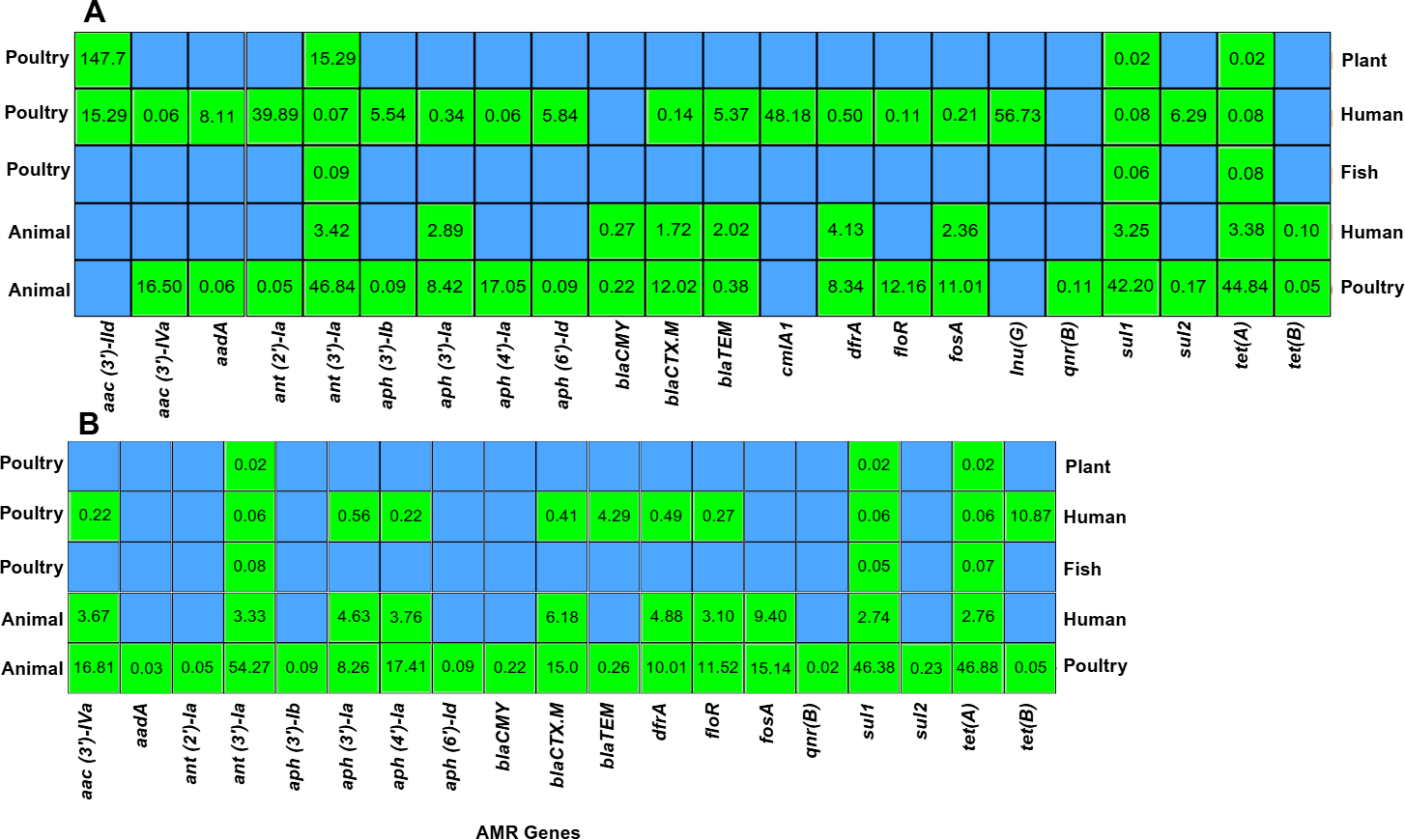
Odds ratio of antimicrobial resistance genes among *S*. Infantis across source. (**A**) Global isolates. (**B**) Isolates obtained within the USA. The heatmap displays the odds ratios (OR) of AMR prevalence between *S*. Infantis isolated from different sources globally. The reference source is shown on the left, and the comparison source on the right, and the list of the ARGs and pESI-like is shown at the bottom. If the OR value > 1, it indicates a higher prevalence of ARGs in the compared source relative to the reference, and OR value < 1 indicates a higher prevalence in the reference source. Green tiles with annotated OR indicate statistically significant pairs (FDR-adjusted *P* < 0.05), whereas blank blue tiles represent nonsignificant comparisons where the FDR-adjusted *P* > 0.05.

The source of isolation of bacteria has a significantly impact on the odds of detecting AMR genes in *S*. Infantis (Fig. 6; File S4). To assess the difference in AMR gene odds ratio among sources (animal, poultry, human, egg, fish, and plant), we performed an analysis considering each source as a reference, ensuring that each source was compared with every other source. Isolates obtained from animal sources had significantly lower odds (FDR-adjusted *P* < 0.05) for ten AMR genes [*aac (3*′*)-IVa, ant(3*′*)-Ia, aph (3*′*)-Ia, aph (4*′*)-Ia, bla_CTX_._M_, dfrA, floR, fosA, sul1,* and *tet(A*)] and had significantly higher odds (FDR-adjusted *P* < 0.05) for ten AMR genes [*aadA, ant (2)-Ia, aph (3)-Ib, aph (6)-Id, bla_CMY_, bla_TEM_, cmlA1, qnr(B), sul2,* and *tet(B*)] compared to poultry. Compared to the animal source, *S*. Infantis isolated from the human source had higher odds for seven AMR genes and lower odds only for two genes [*bla_CMY_* and *tet(B*)]. *S*. Infantis isolated from the human source had significantly higher odds (FDR-adjusted *P* < 0.05) of detecting nine AMR genes [*aac (3*′*)-IId, aadA, ant (2*′*)-Ia*, *aph (3*′*)-Ib, aph (6*′*)-Id, bla_TEM_, cmlA1, Inu(G*), and *sul2]* and lower odds for 10 AMR genes [*aac (3*′*)-IVa, ant(3*′*)-Ia, aph (3*′*)-Ia, aph (4*′*)-Ia, bla_CMY.M_, dfrA, floR, fosA, sul1,* and *tet(A*)] compared to the poultry source. (Fig. 6A; File S4).

Within the USA, there was more variation compared to the worldwide odds ratio of identifying AMR genes among the sources (Fig. 6; File S5). In the USA, ten AMR genes [*aac (3)-IVa*, *ant (3)-Ia, aph (3)-Ia*, *aph (4)-Ia, bla_CTX.M_, dfrA, floR, fosA, sul1, and tet(A*)] had significantly higher odds (FDR-adjusted *P* < 0.05) of detection in poultry compared to animal-sourced isolates, whereas nine AMR genes [*aadA, ant (2)-Ia, aph (3)-Ib, aph (6)-Id, bla_CMY_, bla_TEM_, qnr(B), sul2,* and *tet(B*)] had significantly lower odds (FDR-adjusted *P* < 0.05) of detection in poultry-sourced isolates compared to animals. Similar to worldwide trends, the odds of detecting the AMR genes in human isolates were higher compared to that in animals; however, while comparing with the poultry, human isolates had significantly higher odds (FDR-adjusted *P* < 0.05) only in four genes: *cmlA1, qnr(B*), *bla_TEM_*, and *tet(B*). Other genes like *aac (3)-IVa, ant (3)-Ia, aph (3)-Ia, aph (4)-I1, bla_CTX.M_, dfrA, floR, sul1*, and *tet(A*) had significantly higher odds (FDR-adjusted *P* < 0.05) in poultry. Additionally, isolates collected from the plant source had lower odds for the *ant (3*′*)-Ia, sul1*, and *tet(A*) compared to the poultry source (Fig. 6B). Even though the odds of AMR detection within poultry sources were analyzed, there was no significant difference in odds of detecting any AMR genes between poultry carcass, ceca, egg, and environment (File S6).

### Continental and source-based odds ratios for plasmid types and pESI carriage

**Fig 7:**
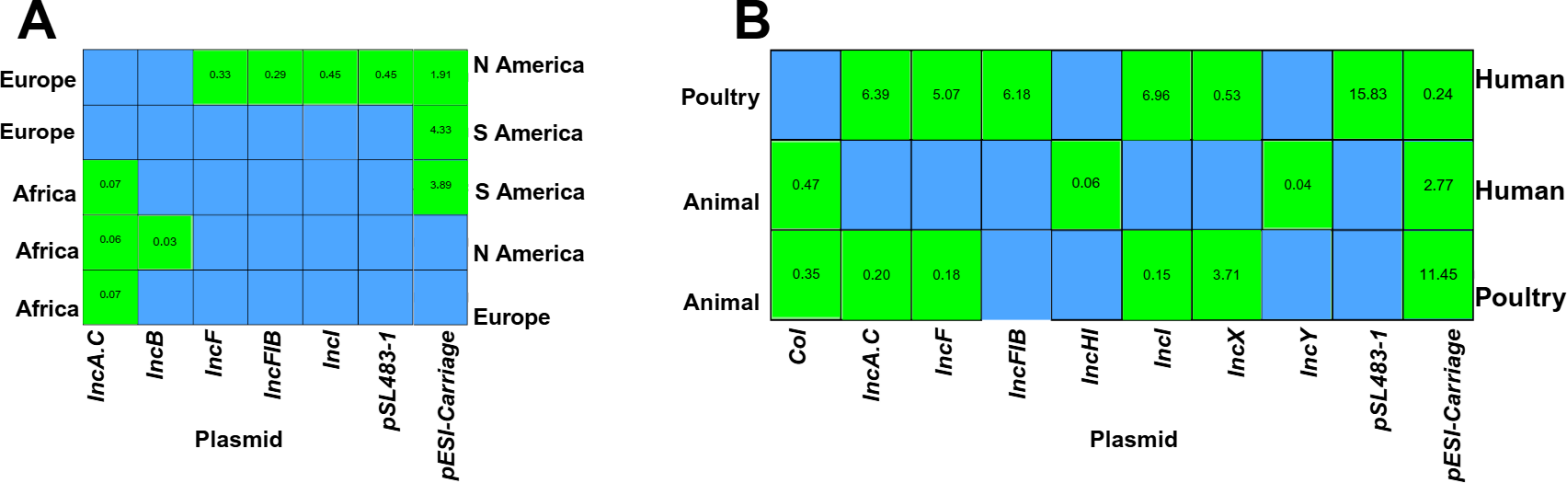
Odds ratio of plasmid types among *S*. Infantis isolates. (**A**) The heatmap displays the odds ratios (OR) of plasmid types between continental pairs (**B**) The heatmap displays the odds ratios (OR) of plasmid types between *S*. Infantis isolated from different sources globally. The reference is shown on the left, the comparison on the right, and the list of the plasmid types is shown at the bottom. If the OR value > 1, it indicates a higher prevalence of ARGs in the compared source relative to the reference, and OR value < 1 indicates a higher prevalence in the reference source. Green tiles with annotated OR indicate statistically significant pairs (FDR-adjusted *P* < 0.05), whereas blank blue tiles represent nonsignificant comparisons where the FDR-adjusted *P* > 0.05.

The odds ratios for acquiring both megaplasmid and other plasmids among *S. Infantis* isolates are summarized in Fig. 7, and the comprehensive raw results from the analysis are provided in File S3. When examining the location-wise distribution of the pESI-like megaplasmid, isolates from South America had significantly higher odds (FDR-adjusted *P* < 0.05) of carriage compared to those isolated in Africa. Additionally, isolates obtained from South America and North America had significantly higher odds (FDR-adjusted *P* < 0.05) of carrying pESI-like megaplasmid compared to European isolates. In contrast, another important AMR gene carrying plasmid IncFIB odds was significantly lower in North American isolates compared to European isolates (Fig. 7A; File S4).

Isolates collected from animals had significantly lower odds of harboring pESI-like plasmids compared to those from poultry and humans, whereas poultry isolates had higher odds than human isolates (Fig. 7B; File S4). In contrast, the odds of harboring other plasmids such as IncA.C, IncF, IncFIB, IncI, and pSL483-1 were significantly higher among human isolates compared to poultry isolates (Fig. 7B; File S5).

## DISCUSSION

Current analysis has revealed several noteworthy findings regarding *Salmonella* Infantis. The predominance of ST-32 *Salmonella* Infantis isolated globally, irrespective of the isolation source—human, animals, plants, fish, and environment—suggests MLST method limitation to identify genomic diversity among the Infantis serovars. This predominance of ST-32 challenges the utilization of the traditional MLST-based approach for bacterial source attribution and epidemiological tracking of *S*. Infantis. We speculate that consistent classification of over 95% of *S*. Infantis as ST-32 might be due to the inability of MLST-based methods to discriminate the evolutionary lineages of *S*. Infantis. Therefore, in order to accurately identify contamination sources during outbreaks, a comprehensive analysis approach such as single-nucleotide polymorphism-based phylogeny could be utilized, which offers higher resolution than MLST-based methods.

We observed that AMR genes associated with key classes of antibiotics, such as aminoglycoside, tetracycline, and sulfonamide, were the most prevalent among the analyzed *S*. Infantis genomes. Among the various locations, isolates collected from South America harbored a relatively higher percentage of these genes as well as had a higher odds ratio, compared to other locations, and Australia had the lowest odds compared to other locations. Although there could be several potential factors that might have caused the variation in the distribution of AMR among *S*. Infantis from the different geographic locations, one major cause might be the usage and access to antibiotics as Australia, Europe, and North America have more strict regulations against the purchase and usage of antibiotics compared to Asia, Africa, and South America, where a prescription is not required for purchasing antibiotics. The lack of regulation on the use of antibiotics in both human and livestock production might have caused higher antimicrobial resistance in certain regions (22). Additionally, the higher prevalence of AMR genes in isolates from Asian and South American regions correlates with the increased abundance of the pESI-like megaplasmid in these regions. Particularly, in our data set, South American isolates clearly exhibit the highest pESI prevalence (84.96%), followed by Asian (74.86%) isolates, compared to Australian isolates showing significantly lower pESI prevalence (11.18%). As the major route for AMR gene transmission occurs via horizontal gene transfer or conjugation between bacteria (23) and *S*. Infantis harbored megaplasmids that can be transmitted between the bacterial species, spreading AMR genes occurs much faster (24). The megaplasmid of *S*. Infantis harbors the genes that allow the plasmid to conjugate (*pil*) (20) and protect from restriction digestion from the host (*ardA* helps production of antirestriction proteins) (25).

Compared to humans, *S*. Infantis isolates collected from poultry sources had higher odds of detecting AMR genes for antibiotics such as cephalosporins, trimethoprim, and tetracycline, whereas human isolates have higher odds for AMR genes related to streptomycin and ampicillin. This result indicates a clear resistance pattern based on the usage of antibiotics. Ampicillin and streptomycin are most commonly used for treating human bacterial infections, and antibiotics such as trimethoprim and tetracycline have been previously used as growth promoters in livestock production. In addition to this antibiotic-specific pattern, AMR genes specific to humans and animals have also been detected in our analysis. We observed that the sulfamethoxazole-specific *sul1* gene was abundant in poultry and *sul2* was abundant in humans. Similarly, chloramphenicol-related genes *floR* in poultry and *cmlA1* in humans and β-lactam-related genes *bla_CMY_*_-M_ in poultry and *bla_TEM_* in humans were observed.

Additionally, poultry carcass isolates exhibited higher odds of AMR genes compared to poultry ceca isolates. This may be attributed to the bacterial stress responses activated during processing, such as heat during scalding and cold during chilling, use of different antimicrobial processing aids and disinfectants, and nutrient limitation, which could potentially enhance bacterial stress tolerance and increase horizontal gene transfer between the bacterial communities. A bacterial stress response against the superoxide stress regulon has been previously found to be associated with enhanced antibiotic resistance in *Enterobacteriaceae* (26).

The pESI-like plasmid carriage rate among *S*. Infantis worldwide and within the USA was much higher among the poultry (87.9% worldwide and 88.3% within the USA) isolates, and the odds of detecting pESI-like plasmids in poultry were higher compared to those in animals and humans. Similar to our study, McMillan et al. (27) also analyzed the carriage rate of the pESI-like plasmid among poultry isolates and cattle isolates from *S*. Infantis isolated between 2017 and 2018 by the USDA-FSIS and obtained the carriage rate for poultry to be 86.2% and that of cattle to be 12.5% (20). However, the odds of detecting the pESI-like plasmid were not impacted by the poultry source. In addition to source variability, the carriage rate and odds of detecting pESI were also significantly impacted by the location, and compared to other continental locations, isolates from South America had higher odds of detecting a pESI-like plasmid. However, the exact reason is currently unknown and requires further research.

One of the major limitations of this study was the disproportionate number of *S*. Infantis isolates between the different sources and locations, and incorporation of the draft genome might have influenced the AMR and plasmid prediction, which might have influenced the statistical and epidemiological power for detecting the prevalence and odds ratios.

### Conclusion

This study provides valuable insights into MLST, AMR, and carriage rate pESI-like plasmid among the *S*. Infantis isolated globally from diverse sources. The results indicate that ST-32 accounts for more than 95% of globally isolated *S*. Infantis. The prevalence of AMR genes among *S*. Infantis is greatly influenced by the location and source of isolation, highlighting the localized niche of AMR transmission and persistence. Additionally, our analysis revealed that compared to other sources, isolates from poultry sources possess a higher prevalence of AMR genes, and odds of detecting AMR genes were also higher among isolates obtained from the poultry sources compared to other sources. Furthermore, the spread of the pESI-like plasmid among the Infantis serovar is worldwide, and the spread of pESI-like plasmids is higher among the poultry isolates, making poultry a major reservoir for pESI-like plasmids. In conclusion, these results highlight the growing global concerns of antimicrobial resistance and the widespread distribution of pESI-like plasmids among *S*. Infantis. The increasing prevalence of these potential MDR *S*. Infantis within the poultry production chain poses a major public health threat, with the further possibility of transmission to humans potentially causing severe invasive infection.

## SUPPLEMENTAL MATERIAL

File S1 spectrum.04020-25-s0001.xlsx Tables S1 to 15.

File S2 spectrum.04020-25-s0002.txt R scripts used for statistical analyses and visualization, including logistic regression models, odds ratio calculations, FDR correction, and heatmap generation.

File S3 spectrum.04020-25-s0003.txt Raw odds ratio results from logistic regression analyses comparing the prevalence of AMR genes, plasmid types, and pESI-like megaplasmids across continents.

File S4 spectrum.04020-25-s0004.txt Raw odds ratio results from logistic regression analyses comparing the prevalence of AMR genes, plasmid types, and pESI-like megaplasmids among global sources.

File S5 spectrum.04020-25-s0005.txt Raw odds ratio results from logistic regression analyses comparing the prevalence of AMR genes, plasmid types, and pESI-like megaplasmids among sources within the United States.

File S6 spectrum.04020-25-s0006.txt Raw odds ratio results from logistic regression analyses comparing the prevalence of AMR genes, plasmid types, and pESI-like megaplasmids among poultry-associated sources.

ASM does not own the copyrights to Supplemental Material that may be linked to, or accessed through, an article. The authors have granted ASM a non-exclusive, world-wide license to publish the Supplemental Material files. Please contact the corresponding author directly for reuse.
